# Review of Dendritic Cells, Their Role in Clinical Immunology, and Distribution in Various Animal Species

**DOI:** 10.3390/ijms22158044

**Published:** 2021-07-28

**Authors:** Mohammed Yusuf Zanna, Abd Rahaman Yasmin, Abdul Rahman Omar, Siti Suri Arshad, Abdul Razak Mariatulqabtiah, Saulol Hamid Nur-Fazila, Md Isa Nur Mahiza

**Affiliations:** 1 Department of Veterinary Laboratory Diagnosis, Faculty of Veterinary Medicine, Universiti Putra Malaysia (UPM), Serdang 43400, Selangor, Malaysia; myzanna6834@gmail.com; 2 Laboratory of Vaccines and Biomolecules, Institute of Bioscience, Universiti Putra Malaysia (UPM), Serdang 43400, Selangor, Malaysia; aro@upm.edu.my (A.R.O.); mariatulqabtiah@upm.edu.my (A.R.M.); 3 Department of Veterinary Pathology and Microbiology, Faculty of Veterinary Medicine, Universiti Putra Malaysia (UPM), Serdang 43400, Selangor, Malaysia; suri@upm.edu.my (S.S.A.); nurfazila@upm.edu.my (S.H.N.-F.); nurmahiza@upm.edu.my (M.I.N.M.); 4 Department of Cell and Molecular Biology, Faculty of Biotechnology and Biomolecular Science, Universiti Putra Malaysia (UPM), Serdang 43400, Selangor, Malaysia

**Keywords:** dendritic cells (DCs), human, animal species, clinical immunology

## Abstract

Dendritic cells (DCs) are cells derived from the hematopoietic stem cells (HSCs) of the bone marrow and form a widely distributed cellular system throughout the body. They are the most efficient, potent, and professional antigen-presenting cells (APCs) of the immune system, inducing and dispersing a primary immune response by the activation of naïve T-cells, and playing an important role in the induction and maintenance of immune tolerance under homeostatic conditions. Thus, this review has elucidated the general aspects of DCs as well as the current dynamic perspectives and distribution of DCs in humans and in various species of animals that includes mouse, rat, birds, dog, cat, horse, cattle, sheep, pig, and non-human primates. Besides the role that DCs play in immune response, they also play a pathogenic role in many diseases, thus becoming a target in disease prevention and treatment. In addition, its roles in clinical immunology have also been addressed, which include its involvement in transplantation, autoimmune disease, viral infections, cancer, and as a vaccine target. Therefore, based on the current knowledge and understanding of the important roles they play, DCs can be used in the future as a powerful tool for manipulating the immune system.

## 1. Introduction

Dendritic cells (DCs) were derived from pluripotent hematopoietic stem cells (HSCs) of the bone marrow. They belong to a group of antigen-presenting cells (APCs) that include B-cells and macrophages. They were originally discovered in 1973 by two Canadian scientists named Ralph Steinman and Zanvil Cohn as a previously undefined cell type in the mouse spleen [1], and eventually known as “dendritic cells” because of their typical features of multiple extended dendritic or pseudopodia-like cytoplasmic protrusions during their maturation stage. DCs work as sentinel cells in the immune system and are found in every part of the body, including the skin, peripheral blood, mucosal surfaces, interstitial tissues lymphoid, and non-lymphoid tissue areas of the body [2]. It has been of paramount importance to study DCs because they are the most potent APCs to be able to present antigens to T-cells, and, significantly, they play a vital role in host immunity by inducing innate inflammatory responses to pathogens, effectively and efficiently priming immature T-cells as well as activating and generating immunological memory T-cells and also the induction of B-cell activation. DCs are also involved in other significant immune functions, such as immune tolerance by maintaining steady-state immune homeostasis through continually presenting tissue-derived self-antigens to CD4^+^ and CD8^+^ T-cells, therefore leading to tolerance against those self-antigens. Additionally, they play a major role in the development of an effective adaptive immune response; therefore, DCs are considered to be the center of the immune system, because they provide an important link between the innate and adaptive immune response. Therefore, the importance of DCs in many aspects of biomedical research and health cannot be overemphasized. Their use in diagnostic and research purposes, such as vaccine production and virus-propagation, and their vital role in several DC-based immunotherapies, such as in cancer therapy, where they have recently been used as therapeutic and monitoring tools, makes DCs an important target in disease prevention and treatment; however, despite many studies on DCs, there is still a dearth of information on DCs in humans and various species of animal, which needs to be fully explored. This necessitates the present review, to ascertain the current status of DCs in human and in certain species of animal. Therefore, this review has elucidated the important aspects of DCs, their role in clinical immunology and their distribution in humans and certain species of animal. Hence, this knowledge will provide an overview for future research and clinical trials.

### 1.1. History and Discovery of Dendritic Cells

The first known report on DCs was made in 1868 by a German medical student, Paul Langerhans [3]. He discovered some different sets of cells in the epidermis with a dendritic appearance, and misinterpreted them as being cells of the nervous system because neurons were the only cells known at the time to have such appearance. Then, in 1973, two Canadian scientists, Ralph Steinman and Zanvil Cohn, discovered an immune cell in the spleen and peripheral lymph node of a mouse with a unique “stellate” morphological appearance and named it a “dendritic cell” because of its myriad filopodial protrusions that extended from the cell body. In 1973, Steinman and Cohn also reported that DCs expressed high levels of major histocompatibility complex (MHC) molecules, which are known to be the primary mechanism of antigen presentation. In 1978, the role of DCs as APCs was confirmed using the Mixed Lymphocytes Reaction (MLR) assay with T-Lymphocytes. MLR is an experiment that determines the primary stimulatory ability of DCs to present antigen using the MHC and its capacity to activate naïve T-cells, which recognize the specific antigen being presented.

### 1.2. The Origin and Anatomical Location of Dendritic Cells

DCs are heterogeneous groups of leukocytes that are found in most body parts, but they have different origins, anatomical locations, and surface markers [4]. DCs in humans and mice originate from HSCs of the bone marrow, which are derived from either lymphoid or myeloid precursors. The phenotypic features and anatomical locations of these two precursors differ, but both the myeloid and lymphoid DCs from humans and murine expressed a high level of CD11c, MHC-II, and costimulatory molecules CD40, CD80, CD83, and CD86. Despite that they can be differentiated based on CD1 markers CD8α and DEC 205 [5]. Regarding the location of DCs, myeloid DCs are primarily located in the spleen marginal zone, whereas the lymphoid DCs are in the T-cell areas of the spleen and lymph nodes, precisely in the periarterial lymphatic sheaths (PALS). Even though both human and murine DCs originated from the same progenitor, their subsets play different roles in the regulation of B-cell activation and the differentiation of T-cells into Th-1 and Th-2, and in terms of the expression of surface markers, myeloid precursors expresses CD14+/CD11C+/CD1− and CD14−/CD11C+/CD1+, whereas the lymphoid precursor expresses CD14−/CD11C−/IL3Rα. The CD14+/CD11C+/CD1− surface markers expressed by myeloid DCs possess high phagocytic as well as an endocytic ability when compared to lymphoid precursors CD14−/CD11C+/CD1+. Moreover, both Langerhans and interstitial DCs are strong stimulators of naïve T-cells, although interstitial DCs also play a vital role in the activation and differentiation of naïve B-cells in vitro.

### 1.3. Types of Dendritic Cell Subsets

#### 1.3.1. Classical DCs (Conventional DCs)

cDCs can be divided into two major subsets, based on the expression of the following surface molecules. These include CD8α^+^ and CD103^+^ or CD11b. In addition, both subsets can be found both in the lymphoid tissues, such as the spleen, lymph nodes, and bone marrow, and in most of the non-lymphoid tissue.

(1) CD8α^+^ and CD103^+^ cDCs

The CD8α^+^ and CD103^+^ cDCs are the best-characterized cDC subsets so far, both in terms of phenotype and gene expression signature [6]. Throughout evolution, they appear to be conserved [7]. The development of CD8α^+^ cDCs and their non-lymphoid counterpart, the CD103^+^ (CD11b-) cDC, is coordinated by the same transcription factors, which include the inhibitor of DNA binding 2 (Id2), interferon regulatory factor 8 (IRF8), basic leucine zipper ATF-like 3 transcription factor (BATF), and the nuclear factor interleukin 3 (NFIL3). Therefore, the deletion of any of these genes will eventually lead to severe developmental defects of CD8α^+^ DCs and also the non-lymphoid tissue CD103^+^ cDCs, but not the CD11b^+^ cDCs [8]. Functionally, the splenic CD8 cDCs are specialized in cross-presentations of exogenous antigens on MHC-I molecules to CD8 T-cells. Additionally, the skin-derived CD103 cDCs have been described to display cross-presentation activity. Furthermore, CD8α^+^ cDCs can also present glycolipid antigens in CD1d context and activating and polarizing invariant natural killer T-cells (iNKT) towards the production of T-helper-1 (Th-1) or Th-2 cytokines [9].

(2) CD11b^+^ cDCs

The most abundant cDCs in lymphoid organs are the CD11b^+^ cDCs, except in the thymus, and they have also been found in non-lymphoid tissues. Currently, CD11b^+^ is defined as heterogeneous and remains less well characterized compared to CD8α^+^ and CD103^+^ DCs. CD11b cDCs, for example, can be further subdivided based on additional surface markers such as CD4 and the endothelial cell-selective adhesion molecule (ESAM), but this segregation does not produce any homogenous population of the CD11b^+^, as observed in massive parallel single-cell transcriptome analysis [10]. The development of CD11b^+^ cDCs is generally controlled by transcriptional factors that include RelB, NOTCH2, RBP-J, IRF2 and IRF4. Importantly, the functional aspects of CD11b^+^ is controlled by IRF4. These functions include their MHC presentation [11] and migration. Due to the heterogeneity of CD11b^+^, the assignment of its specific function has remained challenging. To a certain extent, the function of CD11b^+^ DCs in lymphoid organs is still mostly observed through absences of activities associated with CD8α^+^ DCs. It includes their inability to produce certain unique cytokines such as IL-12, and cross-presentation of antigens. Further characterization of CD11b^+^ cDCs can also be done based on the production of cytokines such as IL-6 and IL-23 [12]. Moreover, the prominent producers of pro-inflammatory chemokines such as CCL3, CCL4, and CCL5 following toll-like receptors (TLR) ligand exposure were shown to be splenic CD11b cDCs. However, the relevance of such activities in vivo remains unclear.

#### 1.3.2. Non-Classical DCs (Non-Conventional DCs)

(1) Monocyte-Derived DCs

Monocyte-derived DCs (moDCs) are also known as “inflammatory DCs”. They arise from the myeloid progenitor and play a vital role in the immune response, because during the onset of infection they tend to provide a pool of APCs that can effectively initiate an adaptive immune response. During inflammation or infection, blood circulating monocytes that are normally known to express receptors for Granulocyte Macrophage-Colony Stimulating Factor (GM-CSF), Macrophage-Colony Stimulating Factor (M-CSF), and Interleukin-4 (IL-4), other differentiation, and chemoattractant molecules tend to invade tissues and are attracted to the site of inflammation, where they are subsequently differentiated into moDCs. However, during an infection, upon entry of moDCs to the site of infection, the moDCs produce TNF-α and an inducible nitric oxide synthase (iNOS), which eventually confers innate protection. Therefore the moDCs are also known as “TNFα, iNOS-producing DCs”, thus, in terms of antigen processing, presentation, and cross-presentation, the moDCs are highly potent [13]. Phenotypically, it is difficult to distinguish moDCs from cDCs because both share similar expression patterns of MHC-II, CD11b, and CD11c. However, one of the indicators of their monocytic past is that the moDCs express CD64 and the Fc-gamma receptor 1 (FCgR1) [14]. In addition, also functionally, the moDCs are similar to cDCs, because both DCs were able to process foreign antigens from tissues and eventually migrate via the lymphatic vessels to the nearest lymph nodes, and present the processed antigens to naïve T-cells. After activation, the lifespan of both DC subsets is similar, as compared to the duration of time the DCs reside within tissues during their immature state.

(2) Plasmacytoid Dendritic Cells (pDCs)

pDCs arise from the lymphoid progenitor. They are found throughout the body, circulating in the blood and peripheral organs, and reside within the lymph nodes, spleen, thymus, and bone marrow (BM). However, the BM-plasmacytoid DCs can differentiate into a conventional DC subset; thus, their population does not seem to develop equally to those of other organs. The pDCs display a distinct morphology and surface phenotype, and possess a highly developed secretory compartment [15]. They are characterized by their round shape and plasmacytoid morphology, and have a resemblance to lymphocytes, while the IL3/CD40L cultured pDCs have a microscopic appearance that is similar to that of moDCs [15,16]. pDCs are generally dormant, but upon stimulation by toll-like receptors (TLR7 and TLR9), they secrete copious amounts of interferon-gamma, which plays a major role in viral and bacterial infections. When they are not stimulated, they can exhibit a tolerogenic potential. pDCs have the potential to act as an APC because they express MHC-II and costimulatory molecules, but their ability to phagocytose dead cells and present cell-associated antigens, and to cross-present exogenous antigen on MHC Class-I has not been established. Moreover, based on the above facts, pDCs are known to play a vital role during inflammation compared to conventional DCs (cDCs), which are more involved in the maintenance of the immunological response.

(3) Langerhans or Epidermal DCs

**Figure 1:**
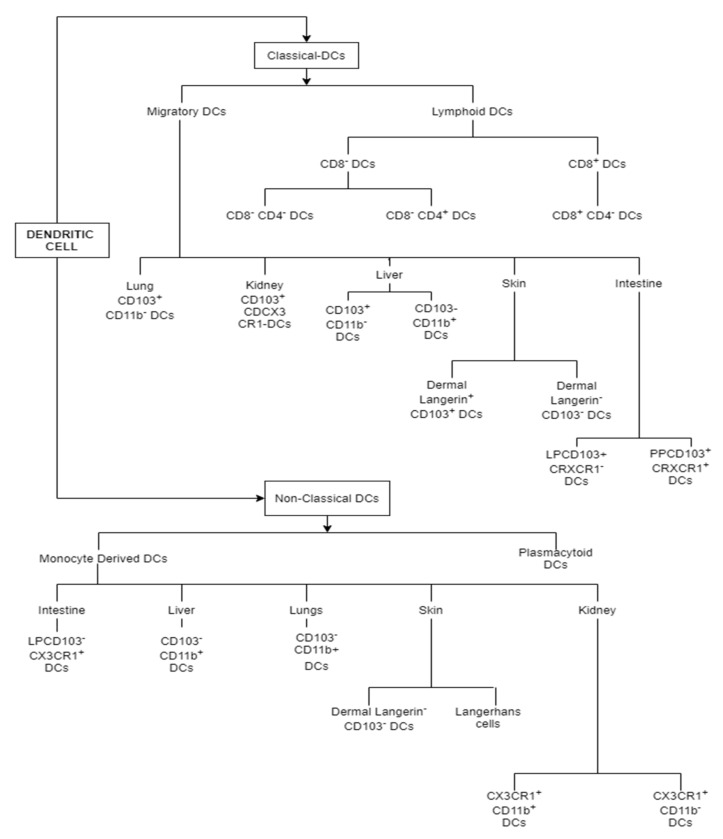
Classification of dendritic cell (DC) subsets as classical and non-classical DCs.

Langerhans cells are a distinct population of mononuclear phagocytic cells that reside within the epidermis. They were the first DC subset to be discovered, in the 19th century, and are considered to be primary sentinel tissue-resident DCs that sample their environment. Upon activation, Langerhans cells (LCs) tend to migrate via the dermis into the nearest regional lymph nodes and eventually present antigens to naïve T-cells. Therefore, this type of DC subset is categorized as “migratory DCs” and other non-lymphoid-tissue-resident DCs, such as dermal and intestinal DCs, also belong to this category of DC subsets. LCs are characterized by the expression of MHC-II, which can stimulate a MLR when cultured in vitro with T-cells. Furthermore, the realization of distinct functional DC “maturation” stages is prompted by studies of LCs. Moreover, LCs are also mainly characterized by the expression of C-type langerin and CD1a, which provides the host with immunity against many skin pathogens, such as bacteria and fungi [17]. LCs also have unique largerin-replete cytoplasmic organelles, known as “Birbeck granules”. Additionally, LCs can also be identified by their expression of monocyte/macrophage and endothelial cell differentiation antigen Ly6C, hence the belief that LCs belonging to the DC family has been clarified by their well-defined gene expression, which is similar to that of macrophages, rather than conventional DCs (cDCs) [18] and particularly, LCs, by their nature, share a prenatal origin with most tissue macrophages. This appears in stark contrast to the short-lived cDCs compartment, which depends on continuous renewal by the HSC-derived cells. Moreover, it is propounded that LCs are more closely related to tissue macrophages than to cDCs, and both cDCs and LCs are necessary required for the maintenance of immunological homeostasis. Their major functions occur during their steady state. The two types of dendritic cell subsets (Classical DCs and Non-Classical DCs) is illustrated below in Figure 1.

### 1.4. The Function and Role of Dendritic Cell in Immunity

**Figure 2:**
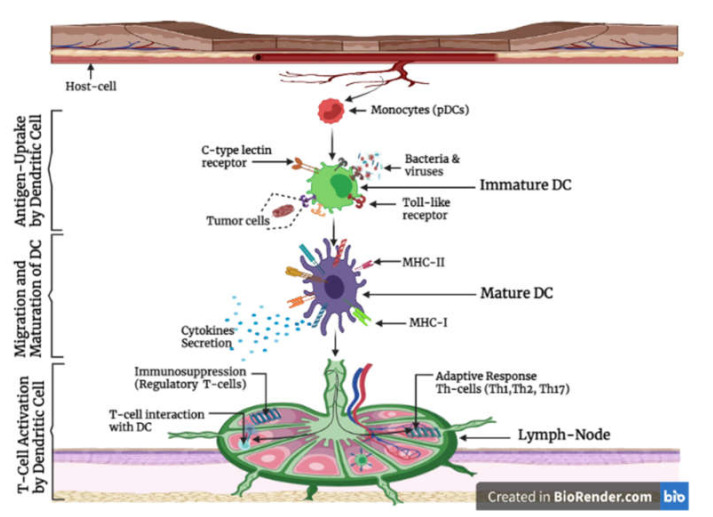
Antigen uptake, migration, maturation and T-cell activation by the dendritic cell.

The function of a DC as an immune cell is to phagocytose, process, and present antigens on the surface of both MHC Class-I and -II molecules to T-cells (Figure 2). The immature DCs, after being produced in the bone marrow via hematopoiesis, will then migrate from the bone marrow and be seeded in the non-lymphoid tissues of the body, where they continuously monitor the extracellular environment for foreign antigens [19]. DCs recognize foreign antigens via specialized receptors on their surface known as “pattern-recognition receptors” such as TLR, C-type lectins (CLR), and intracellular helicases such as retinoic acid-inducible gene-I (RIGI). The receptors detect and bind different types of components on the microbial surface, which is distinct from the host. They bind to a conserved region known as a “pathogen-associated molecular pattern” (PAMP) on the pathogen, similar to cell wall components, such as the lipopolysaccharides (LPS), lipopeptides, or nucleic acids, such as viral or bacterial Ribonucleic-Acid (RNA) and Deoxyribonucleic-Acid (DNA). This triggers the receptors and eventually results in the intake of the foreign particle. The DCs then migrate to the lymph nodes and present the antigens to the T-cells, and eventually the DCs mature, losing their antigen-uptake capacity, and become an APC that possess abilities such as expression of MHC-I and -II on their surface and also up-regulating costimulatory molecules such as CD40, CD80, and CD86 on their surface, and produce large amounts of immunostimulatory cytokines and chemokines. However, the activated naïve CD4^+^ T-cells can differentiate into antigen-specific helper T-cells (TH-cells) of different types upon interaction with the DC, such as Th1, Th2, Th17, and follicular T-follicular helper cells (Tfh-cells), but the types of TH-cell that will be produced depends on certain factors such as the type of antigen captured (bacterial, viral, etc.) and the types of costimulatory molecules and interleukins expressed by the DCs; however, the DCs that reside within the lymph nodes can trap and process antigens in the lymph and present them to naïve CD4^+^ T-cells, to prime and induce the secretion of IL-2, which eventually leads to the proliferation and clonal expansion of the T-cells, playing a vital role in B-cell development and hence antibody production [20].

### 1.5. Dendritic Cells and Immune Tolerance

DCs play a vital role in two types of immune tolerance: central and peripheral [21]. Central tolerance occurs in the thymus. When young T-cells are launched from the thymus, DCs participate in eliminating or deleting those cells bearing “self-reactive antigens or autoreactive antigens” through a process known as clonal selection, before they can harm the body’s own tissues. On the other hand, DCs participate in peripheral immune tolerance, whereby in the absence of infection and inflammation, immature DCs continuously sample and scavenge their environment for cellular debris, dying cells, other self-antigens that cannot access the thymus, or other cells that arise later in life and therefore capture and process those cells, rendering antigens as harmless foreign antigens [22]. Therefore, with the production of IL-10 and activation of the WNT-β-catenin pathway, the DC develops a tolerogenic phenotype, and the tolerogenic DCs migrate into the lymphatic system to present them to naïve CD4^+^ T-cells. Under these circumstances, T-cells specific to self-antigens are induced to differentiate into regulatory T-cells (Treg), or it induces T-anergy or deletion, therefore suppressing or inhibiting the reaction of other immune cells such as the CD8^+^ T-cells, and it eventually prevents the occurrence of autoimmune diseases. Therefore, DCs have a significant role in mediating immune tolerance to their tissues as well.

### 1.6. Cytokine Production

Differentiation of DCs is a process that depends on the presence of some important cytokines, such as Flt3-L, GM-CSF, and M-CSF, and different cell precursors such as the common myeloid and lymphoid progenitors that eventually result in the production of classical (cDCs) and pDCs [23]. Due to the differentiation process, different DC populations acquire different phenotypes as well as residing in different tissues and performing different functions. However, when myeloid and lymphoid progenitors are differentiated in the presence of certain different growth factors, such as GM-CSF and tumor necrosis factor-α (TNF-α), there will be at least two CD populations that are characterized and expressed as CD1a^+^/E-cadherin^+^ and CD14^+^/CD68^+^, respectively, these two CDs populations share some common features, such as the secretion of some cytokines (IL-1, IL-6, IL-7, IL-12, IL-15, IL-18, TNF-, TGF-, M-CSF, and GM-CSF); however, when these cells are induced with CD40 ligands, DCs will then regain the capacity to produce IL-10 and IL-13. The advancement in the maturation and activation of different CD populations brings about changes to the receptors that were expressed on their cell membrane, as well as enhancing their ability to interact with their extracellular environment and other cells.

## 2. The Role of Dendritic Cells in Clinical Immunology

### 2.1. Dendritic Cells in Transplantation

During hematopoietic transplantation, it is the recipient DC that initiates a T-cell induced graft-versus-host reaction, whereas in organ transplantation, both the recipient and the donor DCs contribute to graft rejection. Such condition is known as “graft-versus-host disease” (GVHD), and usually occurs as a result of the recognition of a host alloantigen (along) by donor T-cells, and the condition is the key contributor to the high mortality that is associated with transplantation, including bone marrow transplantation. The residual host APCs initiate GVHD by directly presenting the host antigen (Ag) to donor T-cells. Afterwards, the donor’s APCs mediate antigen presentation, and eventually present the host antigen to donor T-cells via the indirect pathway of antigen presentation, mainly through the MHC Class-II to CD4 T-cells. Therefore, some of the vital roles that the recipient DCs play to overcome such conditions after transplantation are the enhancement of alloimmunity by capturing donor antigens in the graft and eventually activating additional T-cells in secondary lymphoid tissues [24].

### 2.2. Dendritic Cells in Autoimmune Disease

DCs plays a vital role in maintaining a tolerogenic state and preventing the development of autoimmune disease. Therefore, an alteration in either peripheral or central tolerance will lead to the development of autoimmune pathology. Different DC subsets receive several environment signals and, as a result of these signals, there is a wide range of functions, ranging from induction of the periphery to thymic tolerance, to the induction of strong inflammatory responses. Therefore, an introduction of any imbalance in these various DC responses, which result from either complex genetic or environmental factors, would eventually lead to or enhance autoimmune disease. Generally, autoimmune disease is classified into lupus erythematosus (SLE), or tissue-specific autoimmune diseases (such as type-1 diabetes). In SLE, nuclear proteins and self-nucleic acids are usually targeted by the autoreactive B- and T-cell response, and they can be genetically influenced by genes such as human leukocytes antigen loci that play a vital role in determining susceptibility to SLE [25]. In a patient with SLE, pDC and the conventional DC (cDCs) subsets contribute to the onset and severity of the disease, whereby pDCs produce excess interferon-alpha (IFN-α), which in turn activates cDCs that eventually interfere with their ability to maintain peripheral tolerance. However, in psoriasis, which is a common chronic inflammatory skin disorder, the lesional skin is populated with an increased aggregation of natural killer cells, type-3 innate lymphoid cells, TH17 cells, and gα T-cells, which leads to the production of inflammatory cytokines such as the IL-17 and IL-22. This is also observed in type-1 diabetes, which is an autoimmune disorder brought about by defective induction or maintenance of T-cell tolerance towards islet ß-cell self-antigens. In type-1 diabetes, certain genetic and environmental factors contribute to the development of the disease. [26]. In addition, DCs are involved in early diabetes pathogenesis [27], but as a result of the major role DCs play in maintaining Tregs, type-1 diabetes can be blocked by increasing the DC number at the later stage of the disease. DCs that present self-antigens can increase the amount of self-specific Treg, their functions, and their activation, and such types of Tregs that are driven from DCs are potent for inhibiting autoimmunity. However, the steady state of tolerance, which is normally mediated by DCs through either intrinsic genetic alterations or endogenous innate signals that induce some level of maturation, can be disrupted by the autoimmunity.

### 2.3. Dendritic Cells in Viral Infection

DCs have an undetermined role in viral infections, but their propensity to produce a strong cell-mediated immune response and viral clearance from infected tissues is mainly initiated by the ability of DCs to recruit T-cells, produce antibodies from B-cells, and signal other innate immune cells to the site of infection. Moreover, most viral components can activate and promote the production of cytokines, although interactions between the virus and DCs do not always support the host, and these may affect the maturation and functioning of DCs. There are various studies that have reported the interaction of DCs during viral infection. These include herpesvirus, cytomegalovirus (CMV), human immunodeficiency virus (HIV), influenza virus, measles, and respiratory syncytial virus [28]. Impressively, the interaction of DCs with certain viruses produces entirely different outcomes. For example, when a respiratory syncytial virus infects DC, the virus undergoes replication within the DC and allows DC cells to undergo maturation; however, viruses such as influenza, herpesvirus, and dengue can replicate within DC but prevent the maturation process, however, viruses such as the human papillomavirus can present antigens without replicating inside DCs, and HIV exploits DCs to reach its target cell. The autoimmune virus infection of human myeloid DCs inhibits maturation and impairs cross-presentation of antigens via MHC-I [29].

### 2.4. Dendritic Cells in Cancer

DCs play a vital role in the control of cancer by adaptive immunity and via the roles of cytotoxic CD8^+^ T-cells and Th1 helper CD4^+^ T-cells. The foundation of the “cancer immunity cycle” is brought about by the preferential ability of conventional DCs to activate T-cells [30]. Dead neoplastic cells or cellular debris is thought to be endocytosed by tumor-associated conventional DCs and their transport cancer-associated antigens to the draining lymph node, where their prime T-cell and activation can eventually occur. Apart from other DC subsets, other multiple professional APCs also exist, but in terms of initiating T-cell response, directing T-cell polarization, and presenting both exogenous and endogenous antigens on either MHC-I or MHC-II, the conventional DCs were particularly apt for all of these functions. [31]. Basically, there are two main lineages of conventional cDCs (cDC1s and cDC2s) in mice and humans, and they are differentiated by their transcriptional factor dependency, marker expression, and functionality. However, the role of cDC2s in tumor immunity is not well explored. To induce a protective CD8 T-cell, it is necessary to establish a cross-presenting cDC1 population. Using Batf3 mice has convincingly demonstrated this phenomenon, as it fails to reject highly immunogenic cancer cell lines [32] and when using antibodies against programmed death-1, it does not respond to checkpoint blockade therapy [33]. However, despite the significant role DCs play in controlling cancer, tumors are capable of suppressing immunity through their effects on DCs. Cancer-derived cytokines such as IL-6, vascular endothelial growth factor, and IL-10 can suppress DC differentiation and activation.

### 2.5. Dendritic Cells and Targeted Vaccines

DCs are considered to be the major determinants of vaccination, because of the vital role they play in priming T-cell responses against the inoculated antigen. Additionally, they are very important regulatory factors of both humoral and cellular immune responses, which are characterized by immunologic activators and natural adjuvants. These features make DCs valuable in improving vaccine immunogenicity, in enhancement of vaccine efficiency, and in clinical immunotherapy. Presently, one of the most important strategies for improving vaccine immunogenicity in the field of vaccine development is the use of DCs. Designing DC-targeted vaccines has been one of the most extensively studied approaches in vaccine development in recent years, because vaccines induce both humoral and cellular immune responses that eventually induce the clonal expansion of T-cells, thus inducing an effective and durable cytotoxic T-lymphocyte (CTLs) immune response [34].

Currently, there are two types of DC-based vaccines: ex vivo antigen-loaded DC-based vaccines, and in vivo DC-targeted vaccines.

#### 2.5.1. Ex Vivo Antigen-Loaded DC-Based Vaccines

Induction of the protective immune response in humans was first attempted using DCs. It involves the adoptive transfer of in vitro cultured DCs loaded with antigens that are mainly used as immunotherapy against cancer. Bone marrow cells (CD14^+^ cells) that are derived from CD34^+^ progenitor cells and peripheral blood mononuclear cells (PBMC), were cultured in vitro and further differentiated into immature DCs by adding cytokines such as granulocyte-macrophage colony-stimulating factors (GM-CSF) and Interleukin-4 (IL-4) [22]. Afterwards, the immature DCs were activated using stimulus signals to become mature DCs, and eventually loaded with tumor antigen(s) via several methods such as electroporation, active absorption, and adenovirus mediation. DCs loaded with tumor cells or tumor antigens were re-injected into the patient’s body, and migrate to T-cell areas within the lymph organ. Subsequently, they activate T-cells, which eventually stimulate anti-tumor immune responses. The ex vivo antigen-loaded DC-based vaccine is safe and very effective in inducing tumor-specific CD4^+^ T-cells and cytotoxic T-lymphocyte (CTLs) response in humans.

#### 2.5.2. In Vivo DC-Targeted Vaccines

A DC-targeted vaccine is a type of vaccine that targets the antigens, DNA molecules, or drug molecules by identifying certain specific receptors that were expressed on the cell surface, which eventually stimulates the corresponding humoral or cellular immune response. There are several ways of designing DC-targeting vaccines. These include:

(1) Ligand-Based DC-Targeted Vaccines

DCs have many pattern-recognition receptors (PRRs) that are located on their surface, and these PRRs combine specifically with corresponding natural ligands. As a result of this, the conjugation of certain proteins to PRRs-ligands will lead to the generation of antigen–PRR-ligand conjugate vaccines, which ensures simultaneous occurrence of antigen processing and stimulation in the same DCs. During design of an antigen–PRR-ligand conjugate vaccine, there is the need to select appropriate candidates according to the desired immune response, such as Th1-cells, Th2-cells, Th17-cells, and Tc-cell reaction. Thus, DCs can activate and induce clonal expansion of T-cells and at the same time undergo innate immune activation [1].

(2) Antibody-Based DC-Targeted Vaccines

In recent years, the most extensively studied method of activating T-cells has involved the use of antibody-based DC-targeted vaccine, which is mainly due to the exploration of multiple receptor molecules on the surface of DCs cells, and the production of monoclonal antibodies, targeting antigens to DCs in combination with the antibodies. It makes antibodies a favorable delivery vehicle for DC targeting. Most recent antibody-based DC-targeting studies have been geared towards DC acceptor molecules such as C-type lectin receptors (CLRs), integrin and Fc receptors, etc. C-type lectin receptors (CLRs) mainly induce an antigen-specific immune response; therefore, CLRs are capable of internalizing antigens, then delivering them to the endocytic compartments for antigen processing and presentation, which eventually stimulate the required specific immune response. Another DC-targeting acceptor molecule is the Fc receptor, which can interact within the constant region of immunoglobulins. The surface of the DC has many acceptor molecules of immunoglobulins such as IgG, IgA, and IgE, and consists of Fc receptors such as the FcgR, FcαR, and FceR. Of all these Fc receptors [35], the FcgR is involved in many biological processes, which includes cytophagy, immune complex-mediated DC maturation, antigen presentation, and the activation of natural effector cells [36].

(3) Delivery System Based DC-Targeted Vaccines

This involves developing a delivery system for vaccines using adjuvants such as nanoparticles, which ensures that the antigens and the adjuvants can be co-delivered within the same compartment. Therefore, this type of approach will eliminate many disadvantages of the vaccine and will give a promising strategy for vaccine development. In recent years, the development of nanotechnology has made nanoparticles available for delivery of vaccine antigens as well as drugs; moreover, the size and the structure of nanoparticles are considered to be similar to natural pathogens, which enables DCs to take up immune complexes efficiently. In addition, the nanoparticles have many characteristics such as payloads, high signal intensity/stability, and high surface area-to-volume ratio that allows delivery of a high dose of an immunogenic substance within the same vector. Moreover, to maximize a long exposure period of an immune response, nanoparticles can deliver antigens in a slow and sustained manner. Based on the above facts, nanoparticles have become a potential vehicle for the efficient delivery of vaccines [37]. In conclusion, the co-delivery of antigens and adjuvants using suitable vaccine carriers improves the maturation of DCs as well as antigen processing and presentation, which will eventually enhance efficient CD8+ T-cell priming [1].

## 3. Current Dynamic Perspective and Distribution of Dendritic Cells in Human and Various Species of Animal

### 3.1. Human Dendritic Cells

Human DCs are all bone marrow-derived leukocytes, and they originate from a common progenitor, the CD34^+^ hematopoietic progenitor, (Table 1) it gives rise to myeloid (MP) and lymphoid precursors (LP). The MP further differentiates into human monocytes, macrophages, and DC precursors (MDP), which will eventually give rise to monocytes and the common DC precursor [38], the term myeloid is commonly used to describe certain sets of antigen found on conventional DCs (cDCs) [39]. The common DC precursor is mainly found and produced in the bone marrow, where they further give rise to pDCs and a circulating cDCs precursor also known as preclassical DCs (pre-cDCs) that eventually produce two major subsets of cDCs, named cDC1 and cDC2, which are mainly found in the peripheral lymphoid organs [40]. Therefore, based on cytokine-driven conditions, human DCs comprise four subtypes of DC. These are (1) conventional or “myeloid” DCs: CD14− blood moDCs; (2) dermal DCs or interstitial DCs (DDC IDCs); (3) Langerhans cells (LCs); and (4) plasmacytoid DCs. While based on the expression of their surface markers, human blood DCs are divided into three subsets. These are BDCA1 (CD1c), BDCA2 (CD303), and BDCA3 (CD141). It is generally accepted that BDCA1+ DCs resemble mouse CD11b^+^ cDCs; BDCA2^+^CD11c− DCs are equivalent to mouse pDCs; BDCA3^+^ cDCs are equivalent to mouse CD8a^+^ cDCs. Initially, these interspecies associations were based on similarities in gene expression between human and mouse DC subsets, but it was eventually supported by some functional experiments [7]. The identification of human DCs is mainly undertaken by their high expression of MHC Class-II molecules and CD11c, thus phenotypically, their express the following surface markers, MHC-II, CD1c, CD11c, CD141,CD303,CD370,CD123 and morphologically, it appears with an irregular surface with numerous projections and cytoplasmic vacuoles as presented in Table 2. Both are found in other cells such as the lymphocytes, monocytes, and macrophages. Human DCs can be found virtually in all tissues, where they play a major role in homeostatic imbalances and process antigens for presentation to T-cells, thus eventually establishing a link between innate and adaptive immune response. In addition, DCs can also secrete certain cytokines and growth factors [41] that initiate and modify the ongoing immune responses, which will further be influenced by their interactions with other immune cells such as natural killer cells and innate lymphoid cells (ILCs) [42]. Based on location, human DCs can be divided into lymphoid-tissue DCs and migratory, i.e., non-lymphoid-tissue, DCs [43].

### 3.2. Mouse Dendritic Cells

Murine DCs have been categorized into two main lineages: (1) myeloid DCs; and (2) lymphoid DCs. However, mouse DCs can further be subdivided into various subtypes or subsets. Different subsets of mouse DC are classified into:

#### 3.2.1. Resident versus Migratory Mouse DCs

Mouse resident DCs are in secondary lymphoid organs throughout their entire lifespan. Whenever a resident DCs is in a steady state, it displays an immature phenotype and exhibits low levels of costimulatory molecules [44], whereas migratory mouse DCs were present in peripheral tissues and non-lymphoid organs, where they continuously migrate through the lymphatic system to their respective draining lymph node. Thus, they are referred to as “tissue-migratory DCs”. Morphologically, the mouse DCs appears as numerous round cells with an irregular dendritic like projections (Table 2). The initiation of DC migration is induced by cell maturation, which is followed by the up-regulation of MHC-II and other costimulatory molecules in response to certain cytokine stimuli at the steady state and during the process of inflammation [35]. Therefore, resident DCs can only be found in non-draining lymphoid organs such as the spleen, whereas draining lymph nodes possess both resident and migratory DCs, but they can only be distinguished by their unique differential expression of maturation markers [45].

#### 3.2.2. Plasmacytoid DCs (pDCs)

Mouse pDCs are considered to be resident DCs and mainly found in the spleen, bone marrow, thymus, and lymph nodes (Table 1)**.** pDCs display an entirely different feature from other DC subsets but share most functional and morphological features of their human counterparts, such as the CD11c^+^B220^+^Gr1^+^ DCs, which are present in the mouse thymus, and represents the counterpart to IFNα production in human pDCs. pDCs can directly enter lymph nodes from blood vessels. pDCs that are freshly isolated express markers B220 and Gr-1 together with MHC-II, CD8α, CD11c, CD205, CD207,CD40 and FLT3L (Table 2), but lack costimulatory molecules. pDCs are a specialized form of DC that are recognized virus-derived products and hence produce type-interferon [46]. Previous studies have reported that the pDCs are mainly used for antiviral responses; however, they do not play an essential role in antigen presentation [47].

#### 3.2.3. Classical DCs

Classical DCs, also known as the “lymphoid organ resident cDCs”, originated from the pre-cDC progenitor, which stems from the common DC progenitor. They are dependent on Flt3-L; however, they have a short half-life, which needs constant replacement by precursors that originate from the bone marrow. Previous studies using transcriptome analysis have distinguished cDCs from pDCs and other myeloid cell population based on their unique specific molecular signature [18]. cDCs in particular express a specific transcriptional factor known as “zbtb46” [48]. Based on the difference between cDCs ontogeny, this can be further subdivided into two subsets: (1) Batf3-dependent; and (2) IRF4-dependent DCs.

(1) Batf3-dependent DCs consist of resident CD8^+^ DCs and migratory CD103^+^ langerin^+^ DCs. These subpopulations of DCs express some specific receptors, XCR1 and TLR3, and share a common ontogeny and molecular signature [18]. Their development depends on these transcriptional factors IRF8 and Batf3 [6].
(2) IRF4-dependent DCs consist of resident CD8^−^ CD11b^+^ DCs and migratory CD11b^+^. They depend on transcriptional factors RelB and IRF4 for their development, and some unique features of IRF4-dependent DCs include specialization in MHC-II-restricted presentation of antigens after pathogen infection or allergen challenges and the induction of Th17 or Th2 in response to the draining of lymph nodes [49].

#### 3.2.4. Langerhans Dendritic Cells (LCs-DCs)

Mouse LCs-DCs comprise 3–5% of all cells in the epidermis. They express a high level of CD207 (Langerin), CD11c, CD40, and MHC-II, but a low level of CD8. They do not express CD1a, and there is the presence of cytoplasmic Birbeck granules. Contrary to human LCs, murine LCs also express monocyte/macrophage markers such as CD11b of F4/80. The development of murine LCs-DCs do not depend on Fms-related tyrosine kinase-3 (FLT3 or FLT3L) in vivo, as in the case of other DCs subsets, but they share dependency for macrophage-colony-stimulating factor receptor (M-CSF-R) with macrophages [50].

### 3.3. Rat Dendritic Cells

Rat DCs can be studied from two perspectives, in vivo and in vitro.

#### 3.3.1. In Vivo

Rat DCs have been previously isolated in vivo by direct cannulation of afferent lymphatics. This demonstrates the existence of two subsets, the CD4-/SIRPα and CD4^+^/SIRPα (signal regulatory protein), trafficking in the lymph from the small intestine of rats. In the rat, the stage of DC development may be related to the level of CD4 and SIRPα expression. In addition, cells that express CD4^+^/SIPRα are more potent in MLR stimulation but are excluded from T-cell areas. DCs present in the rat intestinal lymph have an irregular morphology with irregular outlines and long cell processes (Table 3), it expresses surface molecules such as CD11c and α€-integrin. In addition, lymph DCs that are freshly isolated and are in a steady state express high surface molecules such as MHC Class-I^high^ and II^high^, and ^low^, CD80^low^, and CD86^low^. The rat splenic DCs CD4/SIRPα, when stimulated with CD40L, produces IL-12, but cells that have the immunophenotype CD4/SIRPα do not. pDCs in rats have been described to have similar immunophenotype and functions to their human and murine pDC counterparts [51].

#### 3.3.2. In Vitro

The basic requirements for the generation of mature DCs from rat bone marrow involves the use of GM-CSF and IL-4 (Table 1). The generation of DCs from rat bone marrow using GM-CSF and IL-4 induces the development of mature DCs that express high levels of surface molecules such as MHC-I and II, CD54, CD11c, CD45, CD80, and CD86 (Table 3). To generate highly enriched and fully mature rat bone marrow DCs, it is necessary to stimulate the DCs with pro-inflammatory TNF-α, bacterial products (LPS), or T-cell contact (CD40L). Additionally, previous studies have shown that the stimulation of mature rat bone marrow DCs with either LPS, CD40L, and/or TNF-α induces the production of IL-12 [52]. Moreover, cells stimulated with LPS (but not with CD40L) secrete TNF-α into the supernatant. Rat monocyte DCs migrate via lymphatic channels to reach the T-cell areas of their respective lymph nodes, in a similar pattern to that of isolated LCs or spleen DCs [53].

### 3.4. Avian Dendritic Cells

In 1978, two scientists, Oláh and Glick, first demonstrated the presence of avian DCs as secretory cells found in the medulla within the bursa of Fabricius and later found in the germinal centers of cecal tonsils of chickens [54]. Chicken DCs have also been isolated and characterized from DC progenitors such as the bone marrow and follicular DCs of secondary lymphoid organs, which include the spleen, harderian glands, Payer’s patches, cecal tonsils, and LCs [55], and all these cells express a unique form of protein known as the “S-100 protein” which is mainly used as an identification marker of the chicken DCs. Additionally, avian follicular DCs have been demonstrated in vivo in the spleen of chickens, where they exhibit an irregular morphology and are divided into two types of cells, the first cell showing a filiform cell process and the second having beaded dendrites (Table 2). On the other hand, Langerhans-like cells in the epidermis of chickens exhibit an ATPase positive reaction and express an MHC Class-II marker, and display intracytoplasmic organelles similar to Birbeck granules of mammalian LCs. They are also found to be present in the cornea, tongue, and esophagus. Previous studies have also reported that ATPase+ epidermal DCs also express CD45 and vimectin, which is similar to mammalian LCs but opposite to keratinocytes [56]. In chickens, the various DC subtypes originate from HSC progenitors but, contrary to this, another study has proved that epidermal DCs could also be derived from yolk-sac-derived erythro-myeloid progenitors as indicated in Table 1 [57]. Avian DCs are located in almost all tissues of the chicken body, which has become necessary for adequate contact with antigens. These tissues/organ surfaces include mucosal surfaces, interstitial tissues, skin epidermis, peripheral blood, and non-lymphoid tissue, but lymphatic tissue has been crucial for DC migration and antigen-presenting functions. Thus, there are several specific markers for chicken DCs that have been discovered via in vitro studies that involve the characterization of chicken DCs. Colony-stimulating factor 1 receptor (CSF1R), which is mostly expressed by macrophage progenitor, monocytes, and chicken DCs, has become the major target for the granulocyte-macrophage colony-stimulating factor, which is necessary for proliferation and differentiation [58]. One way to distinguish monocytes from other myeloid cells, including heterophils and thrombocytes, is by constitutive expression of the CSF1R receptor [59]. DC surface markers such as MHC-II^+^, CD11c^+^, CD40^+^, CD11^+^, CD86^+^, CD83^−^, and DEC-205 (Table 2), were normally expressed by an immature bone marrow-derived DC and they also expressed chemokine receptor CCR6. Immediately after stimulation of the CCR6 receptor, chicken DCs migrate to sites of antigen entry and, after stimulation by the antigen, the DC becomes mature and eventually acquires the ability to present the antigen to the T-cells. The maturation of chicken DCs relies on the up-regulation of CCR7 and the down-regulation of CCR6 [60].

### 3.5. Dog Dendritic Cells

Canine DCs were first discovered in 1985 [61]. The DCs are isolated from canine lymph nodes and blood and have been shown to act as stimulator cells in MLR. They are non-adherent to plastic, and display long “dendritic-like” projections, the isolated canine DCs constantly being contracted and extended. Isolation of canine monocytes DCs has been achieved using elutriation and magnetic microbead purification techniques, and can be generated in vitro from PBMC, and also from bone marrow cells by the separation of CD34^+^ blood progenitor cells (Table 1) [62]. The use of different cytokines in culture medium has been widely used in the subsequent differentiation of the generated cells to canine DCs and stimulation of monocytes with recombinant IL-4, and GM-CSF has been the most widely used approach in generating moDCs [63]. Differentiation of canine DCs from PBMC has been achieved using T-cell-conditioned media without the application of purified cytokines; additionally, previous studies have reported a significant increase in the yield of DCs by up to four times compared to cytokine treatment alone, which has been achieved by the use of human Fms-like tyrosine kinase 3-ligand (Flt3L) in culture medium [64]. Morphologically, cultured canine DCs were found to be non-adherent and displayed a balloon-like “veiled-cell” with variable size and shape, and the cell possesses cytoplasmic projections (dendrites) with large lobulated nuclei (Table 2). Additionally, it has been described in vitro to be bi-nucleated [65]. Phenotypically, canine DCs have a wide range of similarities of cell surface marker expression with human and murine DCs; however, canine DCs express certain unique species-specific markers compared to human and murine DCs. For example, cultured canine DCs do not lose the ability to express CD14, and canine moDCs express high levels of MHC-II and CD11c after seven days of culture [66]. These cultured cells display an increased expression of CD1a, CD40, and CD83, and costimulatory molecules CD80 and CD86 (Table 2) [63].

### 3.6. Cat Dendritic Cells

Feline DCs have not been extensively studied; however, the characteristics and functional properties of feline DCs that have been derived from bone marrow and PBMC using canine GM-CSF and human IL-4 have been described using light microscopy (Table 1). The morphological features that have been observed from the culture of feline DCs reveal a uniformly sized population of non-adherent cells with long cytoplasmic processes and an eccentric nucleus (Table 2), and they express the following surface molecules: CD1a, CD1b, CD1c, CD11c, and CD14, CD18 and MHC Class-I and -II molecules (Table 2) [67]. For feline moDCs, they tend to expressed a high level of CD11b and CD14; however, they lack expression of CD1a, CD1b, and CD1c, and feline moDCs and monocyte-derived macrophages have a similar phenotype, which express high levels of MHC-II, CD1a, and CD80; however, moDCs express low levels of CD11b [68]. The proliferation of lymphocytes in MLR was found to be induced by co-cultured feline DCs with PBMC from allogeneic cats; therefore, these cells were able to induce the proliferation of allogeneic lymphocytes in MLR. Feline LCs were reported to express surface molecules such as MHC Class-II, CD1a, CD4, CD18, and vpg5. The immature stage of feline DCs is mainly related to the high-level expression of CD1, the presence of the mannose receptor, and their ability to phagocytose foreign particles. The mature feline DCs (mDCs) were less capable of up-taking antigens, but they express much larger amounts of MHC and costimulatory molecules on their surfaces, such as the specific marker CD83, and they tend to migrate more efficiently to the lymph nodes [69].

### 3.7. Horse Dendritic Cells

The first equine DCs were isolated and characterized in 1977. The DC was generated ex vivo from a PBMC precursor from domestic animals (horse) using recombinant human rHuGM-CSF and recombinant equine rEqIL-4 (Table 1). Horse DCs have also been demonstrated in-situ in several different tissues, which includes normal and inflamed skin, esophagus, cornea, and lymph node [70]. moDCs in horses have many features that are similar to that of human and murine DCs, displaying a stellate morphology, up-regulating CD86 and MHC Class-I and -II, and rapidly capturing and presenting particulate and soluble antigens, alongside the ability to stimulate CD4^+^ and CD8^+^ viral peptides or alloantigen. If compared with other APCs, moDCs were more potent in terms of stimulating antigen-specific memory T-cell proliferation and cytotoxicity [71]. Equine DCs were phenotypically characterized by high expression of surface molecules such as MHC Class-I and -II, CD86, CD11a, CD14, CD18, CD206 and low expression of CD4 (Table 2). It was found that activators of T-cell proliferation in MLR were ex vivo propagated and allogeneic equine DCs. Therefore, equine DCs were considered to be mature DCs by expression of their phenotypic surface molecules and immunostimulatory activity, while immature DCs were considered by their antigen-uptake activity. Therefore, it is still unclear whether DCs cultured in vitro for 10 days are considered to be mature or immature DCs. Equine Langerhans DCs were characterized to have long dendrites and Birbeck’s granules (Table 2). These cells were usually located in the normal epidermis of the horse and the epidermis junction of equines with cutaneous papilloma. Equine LCs were also previously found in lesions caused by equine insect hypersensitivity; therefore, these cells play an important role in recognizing intake and processing of antigens in this disease [72].

### 3.8. Cattle Dendritic Cells

Bovine DCs are divided into three groups: (1) bone marrow-derived (BMDC/myeloid/conventional-derived DCs); (2) monocyte-related DCs (MoDcs, CD14^+^); and (3) tissue-resident DCs [73]. There are two types of models that are commonly used in isolating and characterizing bovine DCs. These are:

(1) The cannulation of pseudo-afferent lymphatic ducts after surgical removal of the pre-scapular lymph nodes.

This model was originally described in sheep and is now applied in cattle studies. The method allows access to DCs that are draining directly from the skin, and it has not been extensively cultivated in vitro, thus the DCs obtained represent ex vivo DC that are believed to be closely related to the properties of in vivo DCs cells. Therefore, isolation and characterization of bovine DCs are good and well described using the afferent lymph. Morphologically the bovine DCs appears as an irregular cell veiled shape appearance (Table 3). Presently, there are two subsets of bovine DCs that have been described: (i) a major subset, which includes CD11b^high^ CD11a− CD13−, CD26−, CD172^+^, and (ii) a minor subset, CD1b-low, CD11a^+^, CD13^+^, CD26^+^, CD172a^−^ [74]. The bovine DCs that have been isolated from the lymph have been characterized phenotypically and functionally, and one of the major advantages of these lymph-derived bovine veiled-shape DCs cells is their ability to rapidly internalize antigens deposited in the periphery, which will eventually be processed and presented to naïve T-cells in the draining lymph node.

(2) Culture of monocyte-derived DCs in the presence of GM-CSF and IL-4.

This method was originally described in several human studies for DC generation; however, it has been adapted for use in cattle. Recently, monocyte-derived bovine DCs have been generated from experimental and clinical applications using serum-free media [75]. moDCs provide a further valuable model that does not involve the use of prolonged isolation procedures. DCs that are derived from monocytes have been reported to be highly effective at stimulating T-cell response. In uptake, processing, and presentation of antigen, bovine DCs have also been obtained from bone marrow cells cultured with GM-CSF, IL-4 (Table 1), and Flt3-Ligand. Bovine DCs express surface molecules such as CD1^high^, MHC Class II^high^, CD80^high^, CD86^high^, CD11a^high^, CD11b^int^, CD11c^low^, and CD14^low^ (Table 3). Contrary to DCs that have been isolated from the cattle afferent lymph, bovine moDCs express CD14 and CD11b. However, exposing bovine DCs to external stimuli such as the bacterium Salmonella typhimurium increases the expression of surface molecules such as MHC Class-II, CD40, and CD86, compared to macrophages. This shows that when these cells are stimulated with these bacteria, the DCs and macrophages respond differently to infection, and this could have an important implication for the development of the immune response. Previous studies in cattle have reported that the bovine DCs are phenotypically heterogeneous, and that different phenotypes are related to their stage of maturation, which corresponds to different biological properties, and both of the subpopulations of the ex vivo DCs that were isolated from the afferent lymph produces IL-12, IL-6, IL-1, as well as a smaller percentage of interferon-gamma (IFN-γ), while bovine moDCs are capable of producing type-2 cytokines, such as IL-13 and IL-10 [76].

### 3.9. Sheep Dendritic Cells

Ovine DCs can be isolated using two different techniques, which are:

(1) Lymphadenectomy

This technique involves the collection of pseudo-afferent lymph from prefemoral lymphatic vessels. It is also used to yield populations of DCs draining from tissues in the skin and gut [77]. The afferent lymph-derived ovine DCs were characterized based on high expression of MHC Class-II, CD80, and CD86 [78], and cross-reactivity with monoclonal antibodies to the human DC markers CD83 and CMRF-56. Thus, the afferent lymph-derived DCs mainly express high levels of MHC Class-I and -II molecules, CD1 and CD58.

(2) In vitro generation of ovine DCs from adherent peripheral blood mononuclear cells (PBMC) using GM-CSF and IL-4

The ovine DCs were derived from bone-marrow leukocytes and they originate from the common CD34^+^ hematopoietic progenitor, as indicated in Table 1. Morphologically, these cells appear loosely or non-adherent after three days of culture, and have a veiled or dendritic-like appearance (Table 3) [79]. This type of DC expresses CD11c and a weakly mannose receptor. Upon stimulation with LPS, DCs express intermediate levels of MHC Class-I and -II, which is one of the characteristics of immature DCs. The monocyte DCs of sheep are more efficient in terms of presentation of antigens to lymphocytes; therefore, they are regarded to be four to five times more potent than PBMCs. Phenotypically, ovine monocyte DCs express markers such as the MHC-I and II, CD14^+^, CD11b^+^,CD58^high^ CD11c^low^, CD40^low^, CD80^low^, and CD86^low^ (Table 3); however, the CD14 and CD11b are down-regulated during differentiation of the blood cell to moDCs [80]. The subset of ovine DCs consists of the major putative conventional DCs-1 (cDC-1), which includes CD1b^+^, CD26^−^, CD172a^+^, CD205^+^, CADM1^−^, and the minor conventional CD2 (cDC2), which is made up of CD1b^+^, CD26^+^, CD172a^−^, CD205^+^, CADM1^+^ cells [81], and for ovine pDCs it expresses surface molecules such as CD4^−/+^, CD11c^−^, CD14^−^, CD45RB^+^, MHC-II^low^, and CD86^−^, and it is also found to circulate both in the blood and to migrate to the afferent lymph [82].

### 3.10. Pig Dendritic Cells

Porcine DCs can be generated from cultured adherent PBMCs and bone marrow cells (Table 1) [83], and they express a similar phenotype after their maturation. The surface molecules that are expressed by the porcine DCs include SWC3 (a pan-myeloid marker), MHC Class-I and -II, CD1, CD14, CD11a, CD11b, CD11c, CD16, CD80, CD86, and p55 fascin (Table 3). At the immature stage, there is low expression of CD14, but after their maturation, expression of CD14 molecules increases. The morphology of porcine-derived DCs under electron microscopy appears to have a large diameter, with pronounced protrusions, micro-villous projections, and abundant multivacuolar and multilaminar vesicles [52]. There are three subsets of porcine PBMC-derived DCs that are expressed by the pan-myeloid marker (SWC3). These are (1) CD4^-^, CD14^+^, CD16^+^; (2) CD4^-^, CD14^-^, which represents the blood-derived DCs; and (3) CD1^-^, CD4^+^, CD14^-^, and NIPC (natural interferon-producing cells) [84]. Phenotypically, the monocyte-derived porcine DCs express high levels of MHC Class-I and -II, CD1, CD11a, CD18, CD11b, CD11c, CD36, and CD86; however, they express a low level of the CD14 molecule. On the other hand, porcine skin-derived DCs were segregated into four subpopulations: (i) Langerhans cells (Lcs); (ii) CD172^−^ dermal DC; (iii) CD163^+^ dermal DC; and (iv) CD163^low^ dermal DC [85], while porcine LCs express a high level of langerin (CD207) and its constituents are more than 50% of skin DCs. They express surface molecules such as CD172a^+^, CD163^-^, CD16^-^, CADM1^+^, CD207^+^, and MHC Class-II^+^ [85]. Porcine DCs have also been discovered and characterized in tonsils and lymph nodes. These organs possess two defined populations: CD172a^+^, CD11R1^+^, CD1^+/−^, CD80/86^+/−^, and CD172a^+^, CD4^+^, CD1^+/−^, CD80/86^+/−^ DCs. These populations of DCs correspond to cDCs and pDCs, respectively. Moreover, another type of DC that has been discovered is the porcine respiratory DCs. These consist of three subsets: cDC1, cDC2, and inflammatory moDCs [86]. All the above-mentioned subsets have a migratory capacity and are also able to stimulate naïve T-cells; in addition, the cDC2 subset expresses two types of surface molecules, FCeR1α and langerin, and are located in or next to the tracheal and bronchial epithelia [86].

### 3.11. Non-Human Primate (NHP) Dendritic Cells

DCs from non-human primate (NHP) models are mostly generated and characterized via in vitro DC studies (from monocytes, PBMCs, and bone marrow cells). The first NHP species in which in vitro propagation of DC was described was the chimpanzee, and DCs have been best described in the rhesus monkey. Other old-world primate (OWP) species in which DCs have been studied include the Chinese rhesus macaque, African green monkey, and baboon, while for new-world primate (NWP) species, they include the owl monkey and the common marmoset monkey [87]. The most attractive source of DCs for NHP research is through in vitro generation of moDCs, as a large number of DCs may be readily generated from precursor cells available in peripheral blood. This method was originally employed in humans, but later adapted for the generation of a large number of DCs from rhesus macaque blood, but in monkeys it involves the addition of a monocyte-conditioned medium (MCM) for the maturation of cells. There are two types of effective techniques that have mostly been applied in isolating monocytes in most NHP species. These are immunomagnetic bead separation and the plastic adherence method. Previous studies have shown that DCs generated via an in vitro study from chimpanzee peripheral blood were found to be closely similar to human DCs in terms of morphology, phenotype, and function. Morphologically, chimpanzee DCs appear to have an irregular shape DCs, with large eccentric nuclei, few visible granules, and prominent long multiple projections or hairy cytoplasmic projections (Table 3). Phenotypically, chimpanzee DCs that were generated in vitro, using rhGM-CSF and rhIL-4, express surface molecules such as MHC Class-I and -II, costimulatory molecules, and CD80, CD86, and CD83, and express adhesion molecules CD11a, CD18, CD50, CD54, and CD58, (Table 3) and, functionally, they stimulate strong allogeneic responses and have an efficient ability to present soluble antigen to T-cells [88]. Previous studies have shown that rhesus macaque monkey DCs that are isolated and characterized from CD34^+^ bone marrow progenitors (Table 1), express high levels of CD1a, CD4, and CD11a surface molecules [89]. In 2002, Mehlhop et al. [90] reported that they also express the surface molecules DC-LAMP (CD208) and DEC-205 (CD205) (Table 3). Moreover, it has been reported that two DC precursor subsets isolated from blood and lymphoid tissues of a rhesus monkey, premonocytoid DCs (CD11c^+^, CD123^−^) and preplasmacytoid DCs (CD11c^−^, CD123^+^), closely resemble those in humans [91].

**Table 1:** Dendritic cell origin, location, and functional characteristics.

Spp of Animals:	Human	Mouse	Avian	Dog	Cat	Horse	Cattle	Sheep	Pig	Rat	Monkey	Refs.
(1) **DC-Origin**	BM-Hp	BM-Hp	BM-Hp	BM-Hp	BM-Hp	BM-Hp	BM-Hp	BM-Hp	BM-Hp	BM-Hp	BM-Hp	[92]
	CD34^+^	CD34^+^	Yolk-sac	CD34^+^			CD34^+^	CD34^+^			CD34^+^	[93]
(2) **DC-Location**	PBMC	PBMC	B.Fabricius	PBMC	PBMC	PBMC	PBMC	PBMC	PBMC	PBMC	PBMC	[55]
	Skin	Lymph.N	Peyer’s	Lymph.N	Mucosal	Epidermis	Lymph	Skin	Thymus	Lymph.N	Skin	[94]
	Lungs	Skin	Patches	Intestine	tissue	Lungs	Skin	Lymph	Skin	Skin	Lymph	[95]
(3) **DC-Functional Characteristics**												
(a) Antigen intake	Yes	Yes	Yes	Yes	Yes	Yes	Yes	Yes	Yes	Yes	Yes	[96]
												[97]
(b) Mannose Receptor	High	Low	Low	Low	High	High	High	Low	High	Low	High	[98]
												[99]
(c) Functional MLR	Yes	Yes	Yes	Yes	Yes	Yes	Yes	Yes	Yes	Yes	Yes	[100]
(d) MoDc-Isolation (GM-CSF + IL-4)	Isolated	Isolated	Isolated	Isolated	Isolated	Isolated	Isolated	Isolated	Isolated	Isolated	Isolated	[101]
(e) LC Identification.	Yes	Yes	Yes	Yes	Yes	Yes	Yes	Yes	Yes	Yes	Yes	[102]
												[103]
(f) Cytokine Secretion by LPS	IL-6	IL-1α	IL-1ß, IL-6	IL-6	IL-10	IL-12p35	IL-1ß	IL-5	IL-1ß	IL-1b	IL-1ß	[104]
	IL-12p40	IL-1ß	IL-8, IL-10	IL-12	IL-12	IL-12p35	IL-10	IL-17	IL-8	IL-4	IL-18	[105]
	TNF-α	vIL-12P35	IL-12ß, IFNγ	IL-17	B7.1	IL-12p70	IL-12p40	IFNγ	TNFα	TNFα	TNFα	[106]

**Table 2:** The Dendritic cell (DC) morphology and phenotype in different species of animal.

**Spp of Animals:**	**Human**	**Mouse**	**Avian**	**Dog**	**Cat**	**Horse**	**Refs.**
**DC-Morphology**	Irregular surface with numerous Projections cytoplasmic vacuoles	Round cells with irregular dendritic Protrusion	Irregular morphology with Filiform cell process with beaded dendrites	Balloon-like veiled-shape with cytoplasmic Projections	Uniform sized cells with long cytoplasmic Process	Stellate-morphology with long dendrites Birbeck’s granules	[107]
							[67]
							[70]
							[65]
**Spp of Animals:**	**Human**	**Mouse**	**Avian**	**Dog**	**Cat**	**Horse**	**Refs.**
**DC-Phenotypes**	MHC-II, CD1c	MHC-II	MHC-II	MHC-II, CD86	MHC-I and II	MHC-I and II	[108]
	CD11c, CD141,	CD8α, CD11c	CD11c, CD40	CD1a, CD1c	CD1a, CD1b	CD86, CD11a,	[109]
	CD303, CD304	CD205, CD207	CD83, CD86	CD8, CD11a	CD1c, CD11c	CD18, CD4	[110]
	CD370, CD123	CD40, FLT3L	DEC205	CD18, CD45	CD14, CD18	CD206, CD14	[75]

**Table 3:** Dendritic cell (DC) morphology and phenotype in different species of animal.

**Spp of Animals:**	**Cattle**	**Sheep**	**Rat**	**Pig**	**NHP (Monkey)**	**Refs.**
**DC-Morphology**	Irregular cells with veiled shapes appearance	Cells with veiled-shape Dendritic-like shape appearance	Round cells with irregular outlines and long cell process	Cells with pronounced protrusions with micro-villous projections	Irregular cells with long multiple projections or hairy cytoplasmic projections	[79]
						[87]
						[111]
**Spp of Animals:**	**Cattle**	**Sheep**	**Rat**	**Pig**	**NHP (Monkey)**	**Refs.**
**DC-Phenotype**	MHC-II, CD11c	MHC-I and II	MHC I and II	SWC3, MHC-I and II	DC-LAMP (CD208)	[74]
	CD11a, CD11b	CD1b, CD58	CD54, CD11c	CD1, CD14, CD11a	DEC-205, MHC-I and II	[112]
	CD80, CD86,	CD11c, CD205	CD80, CD86	CD11b, CD11c, CD18	vCD11a, CD50,CD54	[90]
	vCD13, CD26,	vCD209	CD45, α€Intergrin	CD36, CD80, CD86	CD58, CD83, CD86	[113]

## 4. Conclusions and Future Perspective of Dendritic Cells

DCs are considered to be the most powerful APCs, because they are highly efficient at generating a robust immune response among all APCs. Some of the important roles DCs play in clinical immunology include their role during transplantation, where DCs overcome disease conditions such as graft-versus-host disease (GVHD), also known as graft rejection. It does that by enhancement of alloimmunity through the capture of donor antigens in the graft and eventually activating additional T-cells in the secondary lymphoid tissues. In terms of autoimmune disease, DCs exhibit their crucial role by maintaining a tolerogenic state, therefore preventing the development of any autoimmune disease. In addition, during viral infection, DCs produce a strong cell-mediated immune response which is initiated by the ability of the DCs to recruit T-cells, therefore producing antibodies from B-cells and then signaling other immune cells to the site of infection, which eventually clears the virus from the infected tissue. Moreover, their role in controlling cancer is through adaptive immunity and via the roles of cytotoxic CD8+ T-cells and Th-1 helper CD4+ T-cells. Finally, DCs are considered to be a major determinant of vaccination, because of the crucial role they play in priming T-cell responses against the inoculated antigen. In addition, they are very important regulatory factors of both humoral and cellular immune responses, which are characterized by immunologic activators and natural adjuvants. These features make DCs valuable in improving vaccine immunogenicity, enhancement of the vaccine efficiency, and in clinical immunotherapy.

Regarding the future potential based on the above vital roles DCs play in disease and immunotherapy, they can be used as a strong tool in manipulating the immune system by designing an effective and efficient vaccine against diseases such as cancer, and other related diseases. Alternatively, the DC can be manipulated by developing strategies capable of prolonging both its activation state and life span; thus, it is expected that this approach will realize the full therapeutic potential of DCs as therapeutic agents or as a DC-based vaccine in the treatment of many diseases.

## Data Availability

No new data were created or analyzed in this study. Data sharing is not applicable to this article.

## Abbreviations

APCs	Antigen-presenting cells
BM-Hp	Bone marrow hematopoietic progenitor
BF	Bursa of Fabricius
CD	Cluster of differentiation
cDC	Conventional dendritic cell
DCs	Dendritic cell
ESAM	Endothelial cell-selective adhesion molecule
Flt3L	Fms-like tyrosine kinase-3 Ligand
GM-CSF	Granulocyte-macrophage colony-stimulating factor
HSC	Hematopoietic stem cells
IL	Interleukin
IFN	Interferon
iNOS	Inducible nitric oxide synthase
LCs	Langerhans cells
LPS	Lipopolysaccharide
LN	Lymph node
M-CSF	Monocyte colony-stimulating factor
MHC	Major histocompatibility complex
MLR	Mixed Lymphocyte Reaction
MoDC	Monocyte-derived dendritic cell
PAMP	Pathogen-associated molecular pattern
PBMC	Peripheral blood mononuclear cells
pDC	Plasmacytoid dendritic cell
NHP	Non-human primates
Spp	Specie
SLE	Systemic lupus erythematosus
